# Pharmacokinetics of isavuconazonium sulfate and its active metabolite isavuconazole in healthy dogs

**DOI:** 10.1371/journal.pone.0305766

**Published:** 2024-07-16

**Authors:** Erin McQuinn, Jonathan P. Mochel, David Borts, Andrew S. Hanzlicek, Karin Allenspach, Jean-Sébastien Palerme

**Affiliations:** 1 Department of Veterinary Clinical Sciences, College of Veterinary Medicine, Iowa State University, Ames, Iowa, United States of America; 2 Department of Biomedical Sciences, College of Veterinary Medicine, Iowa State University, Ames, Iowa, United States of America; 3 Department of Veterinary Diagnostic and Production Animal Medicine, College of Veterinary Medicine, Iowa State University, Ames, Iowa, United States of America; 4 MiraVista Diagnostics, Indianapolis, Indiana, United States of America; University of Veterinary and Animal Sciences, PAKISTAN

## Abstract

Invasive fungal infections (IFIs) are growing in importance in veterinary and human medicine. IFIs such as aspergillosis, blastomycosis, coccidioidomycosis and histoplasmosis remain challenging to treat in dogs. Isavuconazole is a novel antifungal medication that, when compared to currently used azoles, has an expanded spectrum of antifungal activity Rudramurthy (2011), Pfaller (2013), Spec (2018), has more predictable pharmacokinetics in humans Desai (2016), Cojutti (2021) and may cause fewer side effects such as liver and renal toxicity Maertens (2016), DiPippo (2018). The pharmacokinetic profile and safety of isavuconazole in dogs has not yet been characterized. The purpose of this study was to evaluate the pharmacokinetics of isavuconazole in healthy dogs that received a single dose of the prodrug isavuconazonium sulfate. Using full crossover design, six healthy beagle dogs received isavuconazonium sulfate at a mean (+/- SD) dose of 20.6 (+/- 2.8) mg/kg orally and 21.8 (+/- 4.2) mg/kg intravenously. Plasma was collected for batched pharmacokinetic analysis of prodrug and metabolite, isavuconazole, by ultra-high-pressure liquid chromatography tandem mass spectrometry (UHPLC-MS/MS). The median (Q1-Q3) maximum isavuconazole peak plasma concentration was estimated at 3,876.5 (2,811.0–4,800.0) ng/mL following oral administration, with a median (Q1-Q3) peak level at 1.3 (1.0–2.0) hours. Following intravenous administration, the median (Q1-Q3) isavuconazole peak plasma concentration was estimated at 3,221.5 (2,241.5–3,609.0) ng/mL, with a median (Q1-Q3) peak level at 0.4 (0.3–0.6) hours. The median (Q1-Q3) half-life of isavuconazole was 9.4 (7.0–12.2) hours and 14.0 (8.1–21.7) hours for oral and intravenous routes, respectively. One dog received inadvertent subcutaneous drug administration without any apparent adverse effects. Another dog experienced an anaphylactic reaction following accidental rapid drug infusion. No other drug-related adverse events were observed. At dosages used in this study, healthy dogs achieved isavuconazole plasma levels comparable to human therapeutic targets, and when properly administered the drug was well-tolerated.

## Introduction

Invasive fungal infections (IFIs) are a growing problem in human and veterinary medicine due to expanding endemic geographic regions, emerging antifungal resistance, and increasing immunocompromised populations [1, 2]. IFIs remain challenging to treat in dogs with current therapies largely based on triazole drugs. Older triazoles have limitations including risk of hepatotoxicity, potential for renal injury with IV formulations and significant cost, and dogs receiving azoles for IFIs may require therapeutic drug monitoring which further increase client costs [3, 4]. Isavuconazole is a triazole antifungal which, compared to older azole drugs, has fewer side effects and less resistance, and it requires no therapeutic drug monitoring in people [5–9]. The pharmacokinetic and safety profile of isavuconazole in dogs has not yet been characterized, which will be needed prior to study or therapeutic use in dogs with IFIs.

Isavuconazole is a potent inhibitor of ergosterol synthesis, disrupting fungal membranes [10]. It is commercially available as the prodrug isavuconazonium sulfate, in an oral as well as an IV formulation with >99% of prodrug converted to active metabolite by plasma esterases in humans [8, 10, 11]. Single dose pharmacokinetics of this drug in humans are characterized by a large volume of distribution (150–494 liters (min-max)), long elimination half-life (56–104 hours (min-max)) and high oral bioavailability [12]. Isavuconazole does not appear to require therapeutic drug monitoring in humans [7]. Moreover, compared to other antifungals in humans, isavuconazole has less inter-patient variability in exposure due to its high bioavailability [13].

Isavuconazole shows an excellent spectrum of activity *in vitro* against a range of invasive molds, yeasts and dimorphic fungi, including resistant strains of *Histoplasma capsulatum* [5, 6, 14]. In 2015, isavuconazole was approved by the FDA for the treatment of invasive aspergillus and mucormycosis in humans [15]. It has been shown to be non-inferior to voriconazole with fewer side effects in people with a variety of invasive mold infections, most of whom were diagnosed with invasive aspergillosis [8]. Isavuconazole has been used to treat histoplasmosis, coccidioidal meningitis and rarer fungal diseases in humans and has comparable efficacy to amphotericin-B in treating human invasive mucormycosis [16–20].

For many drugs, an appropriate canine dose cannot be easily extrapolated from humans because of species differences in bioavailability and systemic clearance, and this may also be the case for isavuconazole. Prior *in vitro* analysis has shown that the conversion rate of isavuconazonium to isavuconazole is considerably slower in dog plasma compared with that in humans, monkeys, or rodents [11]. It is essential to perform pharmacokinetic and safety studies in healthy dogs in order to determine a safe and effective dose before this drug can be used for clinical cases.

The aim of this study was to describe the pharmacokinetics of isavuconazole in the plasma of healthy dogs after oral and intravenous administration of the prodrug isavuconazonium sulfate at a single dose extrapolated from human studies.

## Materials and methods

### Animals

Using a full crossover design, six healthy research beagles were used for this study. Dogs included three castrated males and three spayed females weighing 7.7–10.4 kg at the time of enrollment. Healthy status was established prior to enrollment in each study arm based on lack of clinically significant abnormalities on physical examination, complete blood count and serum chemistry profile utilizing the Procyte Dx Hematology Analyzer and Catalyst One Chemistry Analyzer, respectively (Idexx, Westbrook, ME, USA). All procedures undertaken were approved by the Iowa State University Institutional Animal Care and Use Committee (IACUC-18-1134, protocol 22–089). At study completion, all dogs were housed for use in further research studies unrelated to this one before ultimately being rehomed with individual owners.

### Drug administration

Dogs were randomized to receive a single dose of the prodrug isavuconazonium sulfate either *per os* (PO) or intravenously (IV), following a 12 hour fast, in a full crossover design. Once the initial drug formulation had been administered and following a washout period of six weeks, dogs received the opposite drug formulation in a second arm of the study. The dosage was guided by simple dose adjustment of doses approved for use in humans scaled by bodyweight, but ultimately the dosage was dependent upon limitations posed by dog size and the commercially available capsule size for this drug [21]. The IV formulation was administered through a cephalic or saphenous vein catheter, using an infusion set with an in-line filter, over 1 hour and diluted in 250 mL of 0.9% sodium chloride, per manufacturer recommendations for use in humans [22]. The capsule formulation was given to dogs PO and followed with a 10 mL tap water swallow administered via oral syringe. The prodrug isavuconazonium sulfate was administered at a mean (+/-SD) dose of 20.6 (+/- 2.8) mg/kg PO and 21.8 (+/- 4.2) mg/kg IV.

### Tolerability assessment

Dogs were observed for vomiting, diarrhea, cutaneous eruption, mentation, or attitude change throughout the 1-hour IV drug infusion as well as during timed blood sampling during the first 12 hours following drug administration for both formulations. Temperature, heart rate and respiratory rate were observed prior to drug administration as well as 12 hours later. Complete blood count (CBC) and serum biochemistry were performed prior to and 24 hours following drug administration for each dog. As before, CBC and serum biochemistry profile were analyzed post-drug administration using the Procyte Dx Hematology Analyzer (Idexx, Westbrook, ME, USA) and the Catalyst One Chemistry Analyzer (Idexx, Westbrook, ME, USA), respectively, except where noted.

### Bioanalysis

#### Sample collection

For measurement of isavuconazonium sulfate and isavuconazole in plasma, venous blood samples were collected immediately prior to drug administration as well as at 0.25, 0.5, 0.75, 1, 1.5, 2, 4, 8, 12, 24, 36, 48, 72, 96, 120 and 168 hours following administration [12, 23]. Thereafter, plasma was collected every 168 hours (7 days) until 4 total weeks had elapsed since drug administration. At each time point, 3 mL of whole blood were collected and transferred into EDTA blood tubes. To ensure stability of the isavuconazonium sulfate prodrug with respect to esterase activity, 2 M citric acid (citric acid monohydrate, 99.5+%, Thermo Scientific, Waltham, MA, USA) and 0.1 M paraoxon (Paraoxon-ethyl, PESTANAL analytical standard, Sigma-Aldrich, Milwaukee, WI, USA) were added to blood collection tubes within 24 hours prior to the time of sample collection [11, 12, 23, 24]. Blood samples were centrifuged at 1307 *g* at 4°C for 25 min. Plasma was separated and stored at -80°C until batched analysis of plasma drug and prodrug concentrations.

#### Analytical standard solutions

Isavuconazole and isavuconazonium sulfate analytical standards were purchased from Millipore Sigma (Burlington, MA, USA). Isavuconazole-d4 analytical internal standard was purchased as a 1 mg/mL solution in acetonitrile from Cerilliant Corporation (Round Rock, TX, USA). A stock solution of isavuconazole was prepared at a concentration of 1 mg/mL in acetonitrile using Optima LC/MS Grade acetonitrile from Fisher Scientific (Waltham, MA, USA). A stock solution of isavuconazonium sulfate was prepared at a concentration of 1 mg/mL in 50/50 (v:v) acetonitrile:water using Optima LC/MS Grade acetonitrile and Optima LC/MS Grade water from Fisher Scientific (Waltham, MA, USA). Working stock solutions of isavuconazole and isavuconazonium sulfate were prepared at concentrations of 1, 5, 10, and 20 μg/mL in acetonitrile. A working stock solution of isavuconazole-d4 was prepared at a concentration of 5 μg/mL in acetonitrile.

#### Calibration and quality control standards

Calibration standards containing both isavuconazole and isavuconazonium sulfate at equal concentrations were prepared at concentrations of 0, 20, 40, 60, 80, 100, 200, 400, and 500 ng/mL by spiking appropriate volumes of working stock solutions into blank control canine plasma (Beagle plasma, K2EDTA, gender pooled, 0.2 μm filtered; BioIVT, Hicksville, NY). Citric acid and paraoxon stabilizers were added to aliquots of the bulk blank control canine plasma at the time each aliquot was used. Quality Control (QC) standards containing both isavuconazole and isavuconazonium sulfate at equal concentrations were prepared at concentrations of 30, 150, and 300 ng/mL by spiking appropriate volumes of working stock solutions into blank control canine plasma. Isavuconazole-d4 was added as the internal standard into each calibration and QC standard at a concentration of 50 ng/mL by spiking an appropriate volume of working stock solution. The total volume of all spiking solutions added to each calibration and QC standard was less than or equal to 5% of the total solution volume.

#### Plasma sample preparation

Plasma samples were prepared by protein precipitation. A 100 μL aliquot of plasma was added to a 1.5 mL Eppendorf-style polypropylene tube. 400 μL of Optima LC/MS Grade acetonitrile was added and the tube was capped and manually vortex mixed for 30 s. The polypropylene tubes were next centrifuged at a relative centrifugal force (rcf) of approximately 10,300 *g* for 5 minutes. A 300 μL aliquot of the resulting supernatant was transferred to an HPLC autosampler vial and 300 μL of Optima LC/MS Grade water was added. The vial was capped and manually vortex mixed for 30 s. 5 μL of this prepared sample was injected for LC-MS analysis.

#### LC-MS method

Liquid chromatography–mass spectrometry (LC-MS) analysis was performed using a Vanquish Flex Ultra High Performance Liquid Chromatography (UHPLC) system interfaced with a TSQ Altis triple quadrupole mass spectrometer (Thermo Fisher Scientific, San Jose, CA, USA).

The analytical column was a Thermo Fisher Scientific Accucore C18, 50 x 2.1 mm, 2.6 μm. Mobile phase A was water with 0.1% (v:v) formic acid and mobile phase B was acetonitrile with 0.1% (v:v) formic acid. The column was thermostated to 35°C and the mobile phase flow rate was 0.4 mL/min. The UHPLC gradient started with a composition of 5% B which was held for 0.5 min and then ramped linearly to 100% B at 3.5 min. The composition was held at 100% B for 0.5 min and the step decreased to 5% and held for 1 min to re-equilibrate the column prior to the next injection.

Mass spectral data was acquired in positive ion electrospray mode with the following source settings: spray voltage = 3800 V, sheath gas = 35 arbitrary units (Arb), auxiliary gas = 12 Arb, sweep gas = 1 Arb, ion transfer capillary temperature = 325°C, and vaporizer temperature = 350°C. Both quadrupoles, Q1 and Q3, were operated at a nominal resolution of 0.7 FWHM (full width at half maximum). The CID gas pressure was 2 mTorr.

Mass spectral data was acquired in selected reaction monitoring (SRM) mode. For isavuconazonium, the precursor ion was the doubly charged ([M+2H]^2+^) ion at *m/z* 359.7 and the product ions were *m/z* 438.1, 224.0, and 165.0. The collision energies for each transition were 12, 20, and 16 eV, respectively. The dwell time for each isavuconazonium transition was 53 ms. Signal from all 3 transitions were summed and integrated to yield peak areas used for quantitation. For isavuconazole, the precursor ion was the singly charged ([M+H]^+^) ion at *m/z* 438.1 and the product ions were *m/z* 224.0 and 127.0. The collision energies for each transition were 21 and 52 eV, respectively. The dwell time for each isavuconazole transition was 71 ms. Signal from both transitions were summed and integrated to yield peak areas used for quantitation. For isavuconazole-d4 internal standard, the precursor ion was the singly charged ([M+H]^+^) ion at *m/z* 442.2 and the product ions were *m/z* 373.1 and 210.0. The collision energies for both transitions were 20 eV. The dwell time for each isavuconazole-d4 transition was 71 ms. Signal from both transitions were summed and integrated to yield peak areas used for quantitation. The lower limit of quantitation (LLOQ) was 20 ng/mL.

Study samples that initially gave results for isavuconazole and/or isavuconazonium that were above the upper limit of quantitation (ULOQ) for each compound were diluted and re-analyzed. These samples were diluted either 1:10 or 1:20, as appropriate, with blank canine control plasma containing 50 ng/mL of isavuconazole-d4 internal standard, re-prepared, and re-analyzed.

The LC-MS method was validated with respect to intra- and inter-day accuracy and precision, LLOQ, recovery, and carryover.

#### Pharmacokinetic analysis

A statistical moment (i.e. non-compartmental) approach was used to perform pharmacokinetic analysis of isavuconazonium sulfate and isavuconazole plasma concentrations in commercial software (PKanalix, MonolixSuite 2023R1, Lixoft, France). Assumptions of linearity in isavuconazonium sulfate and isavuconazole pharmacokinetics were made prior to the analysis. Standard PK parameters were generated for both the prodrug (isavuconazonium sulfate) and the metabolite (isavuconazole), as follows:

#### For isavuconazonium sulfate

- Maximum concentration, C_max_ (after PO dosing);
- Time of maximum concentration, T_max_ (after PO dosing);
- Partial area under concentration-time curve estimates from 0 to 2 hr (AUC_0-2_) and 0 to 4 hr (AUC_0-4_);
- Average concentration from 0 to 2 hr (C_avg0-2_) and 0 to 4 hr (C_avg0-4_).

#### For isavuconazole

- Maximum concentration, C_max_;
- Time of maximum concentration, T_max_;
- Total area under concentration-time curve from 0 to infinity, AUC_0-inf_;
- Total area under the moment curve from 0 to infinity, AUMC_0-inf_;
- Partial area under concentration-time curve estimates from 0 to 48 hr (AUC_0-48_) and 0 to 72 hr (AUC_0-72_);
- Average concentration from 0 to 48 hr (C_avg0-48_) and 0 to 72 hr (C_avg0-72_);
- Slope of the terminal phase, computed by linear regression of the logarithmic isavuconazole concentration vs. time curve during the elimination phase, λz;
- Isavuconazole plasma terminal half-life, T_**1/2(λz)**_ = ln(2) ⁄ λ_z_;
- Isavuconazole mean residence time, MRT = AUMC_0-inf_ ⁄ AUC_0-inf_.

Without prior knowledge of the fraction of isavuconazonium that is converted to its active metabolite, pharmacokinetic parameters related to the amount of isavuconazole, such as isavuconazole systemic clearance and volume of distribution, could not be calculated. For data analysis, the first isavuconazonium and isavuconazole concentration post-dose below the LLOQ was inferred to be LLOQ/2, and subsequent data points were excluded from the analysis. A linear/log trapezoidal rule was used to estimate the area under isavuconazonium/ isavuconazole time curves. The slope of the terminal phase λz was derived by linear regression between *Y* (log(concentrations)) and the *X* (time) using a 1/Y^2^ weighting method. Summary statistics on the individual PK parameters were thereafter performed to derive the Q1/Q2 (median)/Q3 quartiles, (min-max) range geometric mean, and harmonic mean when appropriate (i.e., for time-derived parameters).

## Results

### Tolerability assessment

Drug administration was well tolerated though two dogs were excluded from the IV arm of the study due to study related complications. One was excluded due to subcutaneous infusion of the drug rather than IV drug delivery as was intended. Following subcutaneous infusion, no adverse events were noted, and the subcutaneous swelling dissipated within 24 hours without observable cellulitis, necrosis or sloughing following the medication error. A second dog was excluded following an infusion pump error resulting in rapid drug infusion and subsequent anaphylactic reaction. The dog received the IV infusion at a rate twice that of the other dogs. The dog began hypersalivation, vomiting and appeared mentally dull. He was diagnosed with anaphylaxis, upper airway obstruction (with fluid) and aspiration of vomitus. He was treated in the intensive care and markedly improved by 24 hours when he returned to the colony and was excluded from further study. Follow up chemistry analysis was performed 24 hours after the reaction using the Vitros chemistry analyzer (Ortho Clinical Diagnostics, Raritan, NJ) and revealed no abnormalities of any measured analyte.

In dogs for whom PO and IV doses were appropriately administered, no adverse events were observed. Compared to baseline bloodwork prior to drug administration, there were no clinically relevant changes to any of the measured parameters on complete blood count or serum biochemistry for any dog. Select biochemical results prior to and 24 hours following drug administration are given in **Tables 1 and 2**. No hepatic or renal toxicity was documented for any dog with either route of administration.

**Table 1:** Select biochemical values for dogs (N: 6 dogs) before and 24 hours following PO isavuconazonium sulfate administration.

	ALT U/L	ALP U/L	Total bilirubin mg/dL	BUN mg/dL	Creatinine mg/dL
T0h	67.0 (51.0–90.0)	88.5 (46.0–187.0)	0.1 (<0.1–0.2)	14.0 (11.0–26.0)	0.7 (0.6–0.9)
T24h	63.5 (50.0–77.0)	62.0 (33.0–125.0)	0.1 (<0.1–0.5)	24.5 (16.0–33.0)	0.75 (0.7–0.8)
Reference range	10.0–125.0	23.0–212.0	0.0–0.9	7.0–27.0	0.5–1.8

Median (range). Dogs who were excluded from PK analysis are not included. References ranges are for the Catalyst One Chemistry Analyzer (Idexx, Westbrook, ME, USA).

**Table 2:** Select biochemical values for dogs (N: 4 dogs) before and 24 hours following IV isavuconazonium sulfate administration.

	ALT U/L	ALP U/L	Total bilirubin mg/dL	BUN mg/dL	Creatinine mg/dL
T0	74.5 (43.0–96.0)	85.0 (49.0–106.0)	0.15 (<0.1–0.2)	14.0 (9.0–19.0)	0.6 (0.6–0.8)
T24h	82.0 (63.0–103.0)	84.0 (45.0–149.0)	0.15 (<0.1–0.3)	24.0 (23.0–25.0)	0.75 (0.7–0.8)
Reference range	10.0–125.0	23.0–212.0	0.0–0.9	7.0–27.0	0.5–1.8

Median (range). Dogs who were excluded from PK analysis are not included. References ranges are for the Catalyst One Chemistry Analyzer (Idexx, Westbrook, ME, USA).

### Non-compartmental analysis

No dogs had detectable plasma isavuconazonium sulfate or isavuconazole prior to drug administration for either arm of the study. Following drug administration plasma levels were below the LLOQ by 4 hours for isavuconazonium sulfate and 96 hours for isavuconazole in all dogs regardless of route of administration. Therefore, pharmacokinetic data past 4h and 96h were ignored for the statistical moments analysis of the parent drug and active metabolite, respectively.

Descriptive statistics for the pharmacokinetic parameters derived from the non-compartmental analysis are presented in Tables 3 and 4.

**Table 3:** Summary statistics of the plasma pharmacokinetic parameters for isavuconazonium sulfate following a single PO (N: 6 dogs) or IV dose (N: 4 dogs).

RTE	Parameter	min	Q1	median	Q3	max	mean	SD	CV(%)	GeoMean	HarmMean
**IV**	AUC_0-2_ (ng⋅mL⁻^1^⋅h)	390.4	396.0	430.4	544.1	628.9	470.0	110.1	23.4	461.3	
	AUC_0-4_ (ng⋅mL⁻^1^⋅h)	425.3	431.0	465.0	583.8	674.1	507.4	115.1	22.7	498.5	
	C_avg 0–2_ (ng⋅mL⁻^1^)	195.2	198.0	215.2	272.0	314.4	235.0	55.1	23.4	230.7	
	C_avg 0–4_ (ng⋅mL⁻^1^)	106.3	107.8	116.3	146.0	168.5	126.8	28.8	22.7	124.6	
**PO**	C_max_ (ng⋅mL⁻^1^)	21.0	39.5	66.0	81.0	88.0	60.3	28.9	48.0	53.1	
	T_max_ (h)	0.3	0.4	0.6	0.9	1.0	0.6	0.3	51.6	0.6	0.5
	AUC_0-2_ (ng⋅mL⁻^1^⋅h)	15.0	26.0	47.0	58.4	59.7	42.2	20.8	49.2	37.1	
	AUC_0-4_ (ng⋅mL⁻^1^⋅h)	35.0	46.0	67.0	78.4	79.7	62.2	20.8	33.4	59.2	
	C_avg 0–2_ (ng⋅mL⁻^1^)	7.5	13.0	23.5	29.2	29.9	21.1	10.4	49.2	18.6	
	C_avg 0–4_ (ng⋅mL⁻^1^)	8.8	11.5	16.8	19.6	19.9	15.6	5.2	33.4	14.8	

RTE, route; Min, minimum; Q, quartile; Max, maximum; SD, standard deviation; CV%, coefficient of variation (%); GeoMean, geometric mean; HarmMean, harmonic mean. All data were generated using a statistical moment (i.e., non-compartmental) approach in commercial software (PKanalix, MonolixSuite 2023R1, Lixoft, France).

**Table 4:** Summary statistics of the plasma pharmacokinetic parameters for isavuconazole following a single PO (N: 6 dogs) or IV dose (N: 4 dogs).

RTE	Parameter	min	Q1	median	Q3	max	mean	SD	CV(%)	GeoMean	HarmMean
**IV**	C_max_ (ng⋅mL⁻^1^)	1,279.0	2,241.5	3,221.5	3,609.0	3,979.0	2,925.3	1,154.2	39.5	2,695.8	
	T_max_ (h)	0.3	0.3	0.4	0.6	0.8	0.4	0.2	54.7	0.4	0.4
	AUC_0-inf_ (ng⋅mL⁻^1^⋅h)	7,900.9	11,542.3	16,117.8	17,579.5	18,107.0	14,560.9	4,601.6	31.6	13,873.0	
	AUMC_0-inf_ (ng⋅mL⁻^1^⋅h^2^)	153,953.9	160,085.7	174,987.3	191,017.0	198,276.9	175,551.3	19,471.0	11.1	174,741.4	
	AUC_0-48_ (ng⋅mL⁻^1^⋅h)	6,944.2	10,628.2	15,398.4	16,982.4	17,480.3	13,805.3	4761.5	34.5	13,008.8	
	AUC_0-72_ (ng⋅mL⁻^1^⋅h)	7,290.4	10,988.7	15,705.7	17,222.4	17,720.3	14,105.5	4715.5	33.4	13,346.8	
	C_avg 0–48_ (ng⋅mL⁻^1^)	144.7	221.4	320.8	353.8	364.2	287.6	99.2	34.5	271.0	
	C_avg 0–72_ (ng⋅mL⁻^1^)	101.3	152.6	218.1	239.2	246.1	195.9	65.5	33.4	185.4	
	λ_z_ (h⁻^1^)	0.027	0.033	0.054	0.091	0.11	0.062	0.039	62.6	0.053	
	T_1/2(λz)_ (h)	6.1	8.1	14.0	21.7	25.7	14.9	8.7	58.0	13.0	11.2
	MRT (h)	9.3	9.5	11.1	15.8	19.0	12.6	4.5	35.7	12.1	11.6
**PO**	C_max_ (ng⋅mL⁻^1^)	501.0	2,811.0	3,876.5	4,800.0	5,160.0	3,504.2	1,720.7	49.1	2,829.9	
	T_max_ (h)	0.8	1.0	1.3	2.0	2.0	1.4	0.5	39.4	1.3	1.2
	AUC_0-inf_ (ng⋅mL⁻^1^⋅h)	5,528.2	17,106.6	20,569.2	30,502.2	30,705.6	20,830.2	9373.0	45.0	18,293.0	
	AUMC_0-inf_ (ng⋅mL⁻^1^⋅h^2^)	97,921.2	173,511.7	269,271.0	348,880.6	367,284.1	254,356.6	110803.4	43.6	230,218.0	
	AUC_0-48_ (ng⋅mL⁻^1^⋅h)	4,975.5	165,25.9	19,505.0	29,590.5	29,622.3	19,954.0	9196.3	46.1	17,365.0	
	AUC_0-72_ (ng⋅mL⁻^1^⋅h)	5,215.5	167,65.9	20,107.1	300,85.8	30,320.5	20,433.6	9341.1	45.7	17,840.6	
	C_avg 0–48_ (ng⋅mL⁻^1^)	103.7	344.3	406.4	616.5	617.1	415.7	191.6	46.1	361.8	
	C_avg 0–72_ (ng⋅mL⁻^1^)	72.4	232.9	279.3	417.9	421.1	283.8	129.7	45.7	247.8	
	λ_z_ (h⁻^1^)	0.032	0.057	0.074	0.099	0.14	0.079	0.037	46.4	0.071	
	T_1/2(λz)_ (h)	5.0	7.0	9.4	12.2	22.0	10.9	6.0	55.3	9.7	8.8
	MRT (h)	10.0	10.1	11.7	16.1	17.7	12.9	3.2	25.1	12.6	12.3

RTE, route; Min, minimum; Q, quartile; Max, maximum; SD, standard deviation; CV%, coefficient of variation (%); GeoMean, geometric mean; HarmMean, harmonic mean. All data were generated using a statistical moment (i.e., non-compartmental) approach in commercial software (PKanalix, MonolixSuite 2023R1, Lixoft, France).

**Fig 1:**
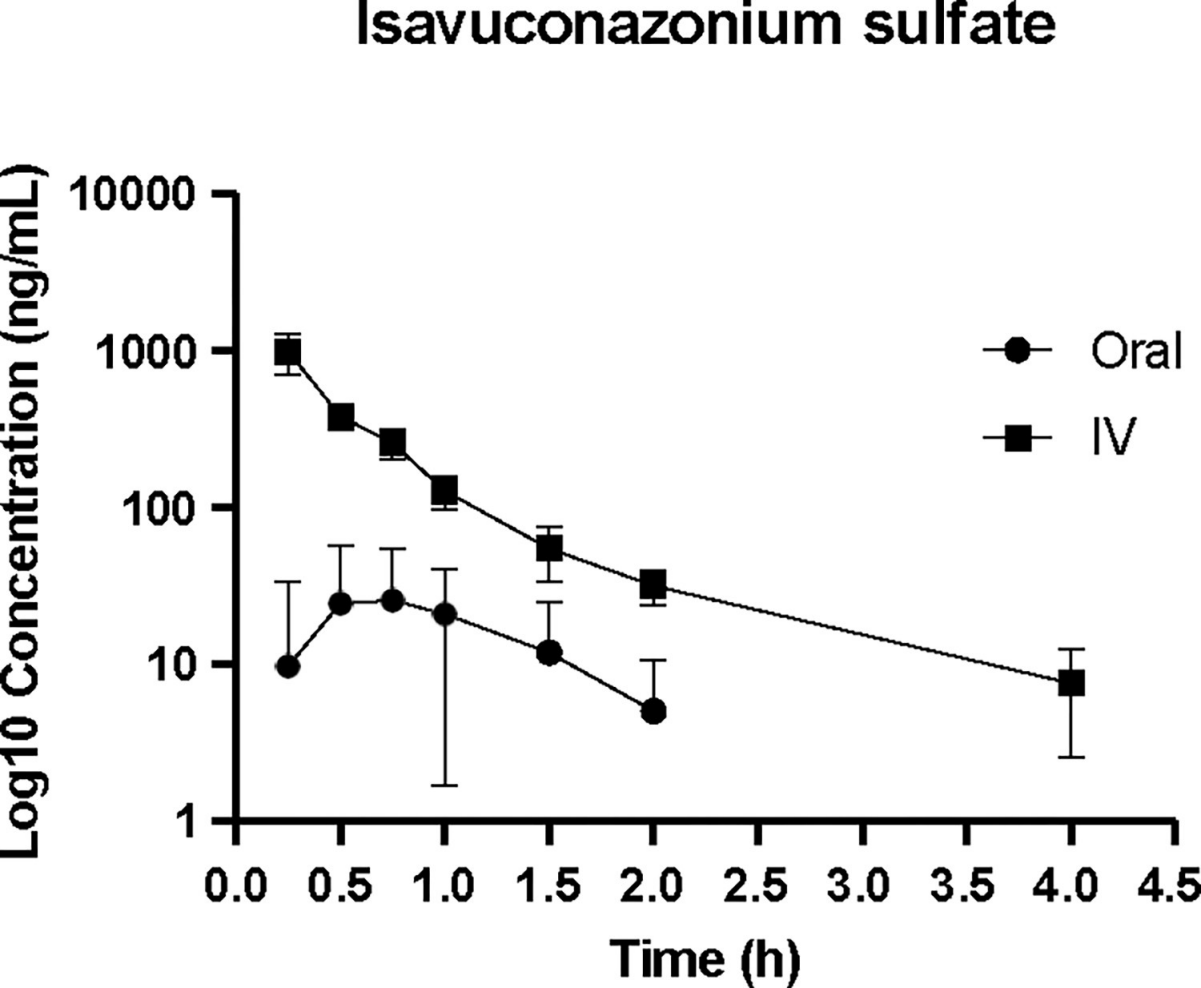
Plasma isavuconazonium sulfate concentrations (ng/mL) over time (hours). Data are presented on a log10 scale with mean and one standard deviation.

**Fig 2:**
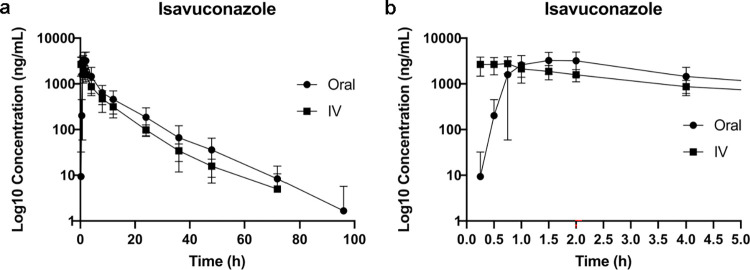
Plasma isavuconazole concentrations (ng/mL) over time (hours). Data are presented on a log10 scale with mean and one standard deviation. **a.** 0–96 hours **b.** 0–5 hours for additional clarity of overlapping data points early in the time course disposition of the drug.

The median (Q1-Q3) maximal isavuconazole peak plasma concentration was estimated at 3,876.5 (2,811.0–4,800.0) ng/mL following PO administration, with a median (Q1-Q3) peak level at 1.3 (1.0–2.0) hours. Following IV administration the median (Q1-Q3) isavuconazole peak plasma concentration was estimated at 3,221.5 (2,241.5–3,609.0) ng/mL, with a median (Q1-Q3) peak level at 0.4 (0.3–0.6) hours. The median (Q1-Q3) half-life of isavuconazole was 9.4 (7.0–12.2) hours and 14.0 (8.1–21.7) hours for PO and IV routes, respectively. **Figs 1 and 2** demonstrate the elimination time course of plasma isavuconazonium sulfate and isavuconazole.

The estimated half-life of elimination of the active metabolite was overall comparable between the two routes of administration, as demonstrated by inspection of the terminal phases of the isavuconazole concentration time course presented in **Fig 2**.

## Discussion

For most healthy dogs in this study, isavuconazonium sulfate appeared to be well tolerated at the administered doses. A notable adverse event was the inadvertent subcutaneous administration of the drug, which caused a transient fluid pocket without appreciable abscessation or necrosis of the involved tissues. Extravasation of drugs is not an uncommon event among veterinary patients. Therefore, the outcome of this inadvertent extravasation of isavuconazole is reported here. One dog experienced an anaphylactic reaction while receiving IV isavuconazonium sulfate at twice the intended rate; whether this reaction was caused by rapid drug infusion or by the drug itself is unknown. For this reason, careful attention should be paid to drug administration rates, and close observation of the patient throughout the administration of this drug is recommended. Prompt cessation of drug administration is advised if signs of an adverse reaction are noted. Preparing doses of diphenhydramine, dexamethasone, and epinephrine ahead of time is also advised until more data is available regarding the true incidence and risk factors for canine anaphylactic reactions to isavuconazole.

Analysis of the active metabolite, isavuconazole, revealed rapid conversion from isavuconazonium, as well as high systemic exposure and relatively rapid systemic elimination. Surprisingly, dogs were able to convert prodrug to drug more efficiently than anticipated. During prior *in vitro* analysis of this drug, conversion from prodrug to drug was considerably slower in dog plasma than in rat, monkey, and human plasma [11]. We therefore hypothesized that conversion to the active isavuconazole metabolite would be inefficient in dogs *in vivo* relative to humans. A couple of dogs had little or no detectable isavuconazonium sulfate prodrug at any point following IV administration, and for most dogs isavuconazonium sulfate was undetectable in plasma by 4 hours after drug administration. Combined with high detectable levels of isavuconazole metabolite, this suggests that conversion of isavuconazonium sulfate to isavuconazole was rapid and efficient in dogs.

Also unexpectedly, isavuconazole was eliminated much more rapidly in dogs than we predicted based upon prior literature in humans. After a single dose of either 100, 200 or 400 mg of isavuconazonium sulfate, the mean +/- SD elimination half-lives of isavuconazole in humans were 63.1 +/- 21.7, 77 +/- 12.8 and 56 +/- 2.49 hours respectively following PO administration and 76 +/- 32.0, 104 +/- 56.7 and 80.4 +/- 33.0 hours respectively following IV administration [12]. By contrast, in this population of dogs the elimination half-life was estimated at approximately 10–15 hours, regardless of the route of administration. Although summary statistics of the elimination half-life of isavuconazole suggests some dissimilarity based on route of administration (median: 9.4 vs. 14.0 hours following PO vs IV dosing, respectively), their estimated ranges were broad and, overall, overlapping suggesting comparable elimination for both routes of administration. This is further supported by visual inspection of the mean isavuconazole time courses (**Fig 2**), which shows that the terminal phases of both dosing routes run in parallel.

In addition to the use of a single breed of dog, another limitation of this study is its small sample size. While the use of 6 subjects is standard for preliminary pharmacokinetic studies, two dogs were excluded from analysis from the intravenous arm of the study due to subcutaneous extravasation of drug and an adverse reaction necessitating intervention. Our experience with inadvertent subcutaneous administration of this drug in 1 dog suggests that this is a minor setback without significant local or systemic consequences. However, this drug has not been studied for subcutaneous administration so whether a patient in this scenario could be dosed again or whether the drug is efficacious if given subcutaneously remain unknown.

The dosages used in this study are relatively higher than those used in human patients. These dosages were selected by design owing to our hypothesis that dogs may be less able to metabolize prodrug into active isavuconazole. Furthermore, selection of drug dosage was somewhat confined by the fixed oral capsule size that was commercially available at the time. A smaller capsule size has since become available. Although therapeutic drug concentrations for isavuconazole have not been established in dogs, this dose provides plasma concentrations comparable to expected therapeutic levels in humans. Finally, maintenance of therapeutic plasma concentrations in humans requires only once daily dosing; based upon dogs’ apparent rapid clearance of isavuconazole in this study, twice daily or more frequent dosing is likely needed to maintain therapeutic drug concentrations in this species.

This study suggests that isavuconazole is well tolerated in healthy beagles when given at these dosages. Additionally, this study suggests efficient conversion of the prodrug to its active metabolite and demonstrates that single doses of isavuconazonium sulfate, administered orally or intravenously, achieved plasma levels within human therapeutic targets in this population. Further studies assessing the pharmacokinetics of isavuconazole in fed dogs, dogs with IFIs, and dogs receiving multiple dosing protocols are needed before it can be recommended for routine use in clinical patients.
